# Occupational biomechanical risk factors for hip and knee arthroplasty incidence: a register-based cohort study in male construction workers

**DOI:** 10.1136/bmjopen-2025-107604

**Published:** 2026-04-06

**Authors:** Kristin Gustafsson, Jens Wahlström, Albin Stjernbrandt, Charlotte Lewis, Sebastian Mukka, Per Liv, Adnan Noor Baloch

**Affiliations:** 1 Department of Health, Medicine and Caring Sciences, Linköping University, Linköping, Sweden; 2 Rehabilitation Centre, Ryhov County Hospital, Jönköping, Sweden; 3 Department of Epidemiology and Global Health, Umeå University, Umeå, Sweden; 4 Department of Diagnostics and Intervention (Orthopaedics), Umeå University, Umeå, Sweden; 5 Department of Public Health and Clinical Medicine, Umeå University, Umeå, Sweden; 6 School of Public Health and Community Medicine, Institute of Medicine, Sahlgrenska Academy at the University of Gothenburg, Gothenburg, Sweden

**Keywords:** OCCUPATIONAL & INDUSTRIAL MEDICINE, Hip, Knee

## Abstract

**Abstract:**

**Objective:**

To evaluate the association between exposure to occupational biomechanical factors and the incidence of surgically treated osteoarthritis (OA) treated with arthroplasty in the hip and knee among male construction workers.

**Design:**

Longitudinal register-based cohort study.

**Participants and setting:**

Male construction workers (n=291 062) who participated in a national Swedish occupational health examination programme between 1971 and 1993, delivered through multiple primary-level nationwide occupational health centres.

**Primary and secondary outcome measures:**

Hip and knee arthroplasties performed due to OA from 1987 to 2019 were identified through linkage with the Swedish National Patient Register. Data on age, smoking habits, body mass index, job title and self-reported biomechanical exposures were collected during the health examinations. Occupational biomechanical workload was assessed using eight factors from a job-exposure matrix. Poisson regression was applied to estimate adjusted incidence rate ratios (IRRs) associated with each type of occupational biomechanical exposure.

**Results:**

The study included 10 336 cases of hip arthroplasties and 8926 cases of knee arthroplasties. All studied biomechanical risk factors were associated with an increased risk of knee OA requiring arthroplasty, especially for individuals exposed to static work in non-neutral lumbar postures (IRR 1.38, 95% CI 1.16 to 1.65) and those with a high frequency of kneeling (IRR 1.27, 95% CI 1.12 to 1.45). In contrast, only a few biomechanical factors were associated with an increased risk of hip OA requiring arthroplasty. Similar results were observed when alternative exposure measures, such as occupational group and self-reported exposure assessments, were employed.

**Conclusions:**

Occupational workload was associated with an increased risk of knee arthroplasty due to OA, whereas the association for hip arthroplasty remains unclear.

**STRENGTHS AND LIMITATIONS OF THIS STUDY**

- A large nationwide cohort of construction workers was linked to inpatient register data with more than 30 years of follow-up, enabling reliable identification of hip and knee arthroplasty due to osteoarthritis.
- Using a job-exposure matrix, the study evaluates biomechanical exposure levels in specific occupational settings and analyses their association with increased risk of hip and knee arthroplasties.
- By including only male construction workers, the study’s findings may not be generalisable to women.

## Introduction

 Osteoarthritis (OA) is a joint disease that primarily affects the hip and knee joints, leading to symptoms such as pain, stiffness and muscular weakness.^1^ It develops gradually, with individuals experiencing varying degrees of symptoms and structural changes. For those with severe OA symptoms and significant structural damage, joint arthroplasty can effectively relieve pain and enhance quality of life.^2^

OA is often linked with ageing but can also affect working-age individuals, with a reported prevalence of 3.5% among those aged 30 to 60.^3^ Additionally, the number of surgeries performed on younger patients is rising.^4 5^ Advances in surgical technique and prosthetic components have contributed to improved long-term durability of hip and knee arthroplasties.^6 7^ The risk of developing hip and knee OA increases with age, particularly due to cumulative exposure to various individual risk factors, such as high body mass index (BMI), but also genetic predisposition and previous knee injuries or hip deformities.^1 8^

Research has also highlighted an association between occupations with high physical demands and hip and knee OA development. Jobs in agriculture, construction, fishing and driving sectors have been noted to carry higher risks of developing these conditions.^9^,^11^ Furthermore, studies have examined how specific work tasks and biomechanical exposures can contribute to the development of OA. For instance, heavy lifting and carrying, non-neutral postures, climbing stairs and whole-body vibration are biomechanical factors associated with an increased risk of developing hip OA. Similarly, activities such as heavy lifting and carrying, kneeling or squatting and frequent climbing have been recognised as specific risk factors for knee OA. Still, there are variations in risk estimates and limited evidence for certain occupational biomechanical exposures, particularly concerning hip OA.^9^,^14^

Järvholm *et al*^15^ studied a large national cohort of construction workers in Sweden from the 1970s to the early 1990s, tracking hip and knee arthroplasties until 1998. They found that individuals in occupations like floor layers, asphalt workers, sheet-metal workers and rock workers faced a higher risk of knee arthroplasty, while the elevated risk for hip arthroplasty was less pronounced. To implement effective preventive measures, it is crucial to understand how different biomechanical exposures in various occupations influence the risk of surgery for both hip and knee OA.^13 15^

Therefore, this study aimed to improve understanding of the association between occupational biomechanical exposures and the incidence of hip and knee OA treated with arthroplasty among male construction workers. Specifically, we extended the follow-up period of the previously studied national cohort of construction workers, evaluated the level of biomechanical exposure in specific occupational settings by using a job-exposure matrix (JEM) and incorporated subjective exposure grading in part of the cohort.

## Methods

### Setting and participants

This longitudinal register-based cohort study followed male construction workers for 33 years to identify arthroplasties in the hip or knee due to OA and to explore the relationship between prior occupational biomechanical exposures and the incidence of surgery. The study is reported according to the Strengthening the Reporting of Observational Studies in Epidemiology guidelines.^16^

The study cohort was selected from a total of 389 132 Swedish construction workers who participated in health examinations as part of a national health surveillance programme conducted from the late 1960s until 1993 (the Construction Workers Cohort). The construction workers were offered free, voluntary health checks through agreements established between employers and labour unions, with approximately 80% completing at least one health examination.^17^

**Figure 1:**
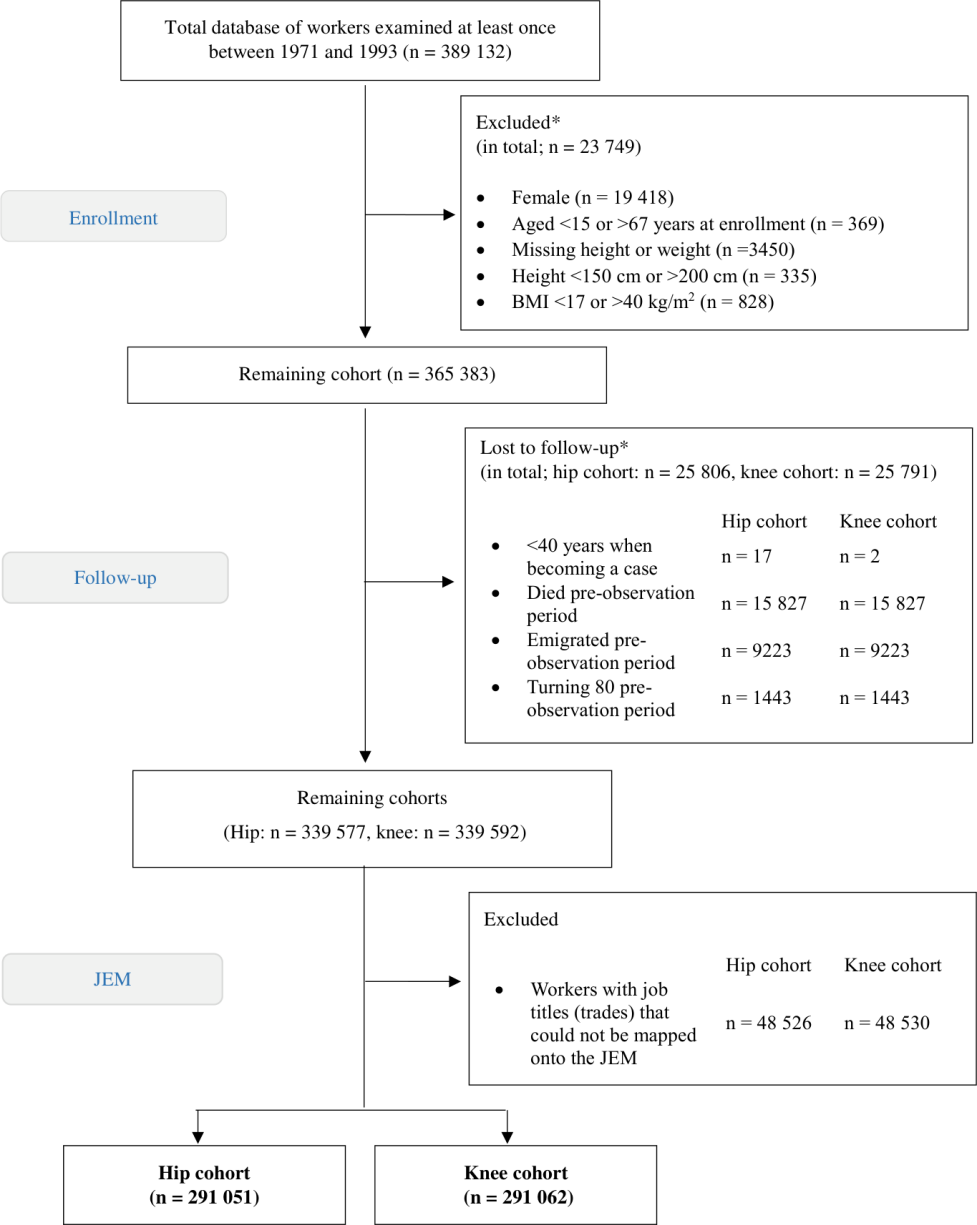
Flow chart of the total study cohort. *An individual may simultaneously meet multiple exclusion criteria or have several reasons for being lost to follow-up. BMI, body mass index; JEM, job-exposure matrix.

In the study cohort, we included male construction workers examined between 1971 and 1993. We used data from each individual’s first health examination, including those aged 15 to 67. Individuals with missing height or weight data, those who were unusually short (<150 cm) or tall (>200 cm), and those with a BMI outside the range of 17 to 40 kg/m^2^ were excluded. Additionally, female workers were excluded as they represented only 5% of the total population and were predominantly employed in white-collar roles or lacked data on job title. Those boundaries aligned with Järvholm *et al*^15^ except that BMI was raised from 35 kg/m^2^, since operating on individuals with higher BMI is performed with effect on patient-reported health-related quality of life.^18^ Furthermore, individuals not able to classify in a specific occupational group were excluded, since they could not be mapped according to the JEM (figure 1).

### Variables

#### Individual factors

Data on smoking habits (self-reported—classified as never, ever or unknown) and height and weight, from which BMI was calculated, were collected from each individual’s initial health examination. Age at the health examination was also recorded.

#### Biomechanical exposures

The job title was collected from their last registered health examination. Based on the occupational codes applied in the Swedish construction industry at the time of these health examinations, 212 job titles have previously been mapped into 21 occupational groups, defined by occupational health service experts at the time of the national health surveillance programme. The occupational groups comprised jobs with similar educational backgrounds and tasks performed. There was also a group for jobs that could not be classified. Full details of the mappings between jobs and their categories have been published previously (online supplemental Table A1-3).^19^

Each of the 21 occupational groups was assigned different levels of occupational biomechanical exposures using a JEM developed for the Construction Workers Cohort. The JEM contained 25 factors, where two experts independently assessed the average exposure intensity or frequency experienced by an individual based on a full-day biomechanical exposure for each of the 21 occupational groups. In the JEM, we identified eight factors of specific interest to study their association with OA based on previous research.^910 12^,^14^ The variables were ‘time spent kneeling’, ‘frequency of heavy lifting up to 25 kg’, ‘heavy loading of the back—not necessarily related to lifting’, ‘frequency of repetitive lifting >25 kg’, ‘frequency of extreme trunk postures’, ‘frequency of static work in non-neutral lumbar postures’, ‘frequency of climbing stairs and ladders’ and ‘magnitude of whole body vibration’, with each rated on a three-point scale between 1 (low/none) and 3 (high) (online supplemental Table A1).

#### Self-reported biomechanical exposure

Between 1989 and 1993, an additional questionnaire was used during the health examinations to gather data on self-reported exposure to various biomechanical exposures. For this study, three questions from the questionnaire were selected: ‘Regarding your working positions. How much do you work: (a) with heavy lifting, (b) in non-neutral working postures and (c) kneeling?’ Each question offered five response options, ranging from ‘rarely’ to ‘often’. Of the total study cohort, 87 105 individuals had responded to at least one of the questionnaires (online supplemental Figure A1).

### Outcome

Two outcome variables were used in this study: the first occurrence of surgically treated hip OA and knee OA, each assessed separately during the follow-up period from 1987 to 2019. Each individual’s diagnosis and surgery codes were identifiable due to the linkage of the Construction Workers Cohort with the Swedish National Patient Register maintained by the National Board of Health and Welfare.^20^ This government-administrated register has compiled nationwide data on all inpatient care in Sweden since 1987. The linkage was made using each individual’s unique personal identity number, which is assigned to all Swedish residents at birth or on being registered as a permanent resident after immigration.^21^

A case was defined as having a main diagnostic code of OA and a surgical code for arthroplasty in the same joint. For hip arthroplasty, the following codes were used: the International Classification of Diseases (ICD)-9 code 715B (applicable between 1987 and 1996) or the ICD-10 codes M16.0 and M16.1 (used between 1997 and 2019). These were paired with the surgical codes 8414 (used between 1987 and 1996) or NFB29, NFB39, NFB49 and NFB99 (applicable between 1997 and 2019) during the same follow-up period. Similarly, for knee arthroplasty, cases were identified using the ICD-9 code 715B or the ICD-10 codes M17.0 and M17.1, in conjunction with the surgical codes 8423, 8424, 8426, 8427 and 8428, or NGB09, NGB19, NGB29, NGB39, NGB49, NGB53 and NGB99.

### Statistical analysis

Poisson regression analyses with a log-person time offset were conducted, designating biomechanical exposure as the primary independent variable and hip arthroplasty due to OA as the outcome variable. The follow-up period began in the year after the initial health examination, on 1 January 1987, or in the year the individuals reached 40, whichever occurred later. Observations were censored at instances of death, emigration, reaching the age of 80 years, or at the conclusion of the study period on 31 December 2019.

A Lexis expansion was applied to split each participant’s follow-up into consecutive 1-year intervals. Age and calendar year were recalculated and updated at the start of each interval. These variables were treated as time-varying covariates in the Poisson regression models, accounting for the time-varying effects of age and calendar year throughout the study period. Poisson regression models were used to calculate incidence rate ratios (IRRs), wherein the counts of hip arthroplasty due to OA and person-years at risk were compared across levels of biomechanical exposures. White-collar workers were the reference category for all models. Incidence rates and IRRs, along with 95% CIs, were reported.

All regression analyses were adjusted for age at follow-up and BMI at health examination (both modelled using restricted cubic splines with four knots positioned at the 5th, 35th, 65th and 95th percentiles), smoking status and the calendar year as a linear effect. Poisson regressions were performed for each of the biomechanical exposures derived from the JEM and for the self-reported biomechanical exposures (among the subgroup that responded to the questionnaire).

All analyses were repeated using knee arthroplasty as the outcome. All statistical tests were two-sided, with level of significance set at 5%. All statistical analyses were conducted using Stata V.18.5 for Windows (RRID: SCR_012763).

### Patient and public involvement

Patients or members of the public were not directly involved in the design or conduct of this specific study. However, the data were derived from the Construction Workers Cohort, an occupational health service originally established and governed through a partnership between labour unions and employers in the Swedish construction industry.

## Results

The sample included 291 051 individuals in the hip cohort and 291 062 in the knee cohort (figure 1). The hip and knee cohorts were nearly identical, except for 15 additional individuals in the knee cohort. The mean age at health examination was 34.0 years (SD 12.6), with a mean BMI of 24.1 (SD 3.1) kg/m^2^, and 53% (n=155 148) self-reported as ever smokers.

The follow-up period, from 1987 to 2019, included 10 336 cases of hip arthroplasties performed due to OA, resulting in an IR of 166 cases per 100 000 person-years (95% CI 163 to 169). In the knee cohort, we identified 8926 cases of knee arthroplasties during the same follow-up period, leading to an IR of 143 cases per 100 000 person-years (95% CI 140 to 146). The mean age at the time of surgery was 65.8 years for the hip cohort and 67.1 years for the knee cohort.

### Associations with JEM-based biomechanical exposures

In the hip cohort, analysis of the eight biomechanical exposures revealed no consistently elevated IRR for hip arthroplasty due to OA. Additionally, there was no clear exposure-response pattern identified between the level of biomechanical exposure and the risk of surgery among individuals with high exposure (see table 1 for adjusted IRR and online supplemental Table A4 for crude IRR). In contrast, the analysis of the knee cohort indicated an increased risk of knee arthroplasty due to OA across all biomechanical exposures. The highest risks were observed among individuals with high exposure to static work in non-neutral lumbar postures, IRR 1.38 (95% CI 1.16 to 1.65), and high frequency of kneeling, IRR 1.27 (95% CI 1.12 to 1.45). Indications of exposure–response trends were observed for a majority of the biomechanical factors except for heavy loading of the back and whole-body vibration (see table 2 for adjusted IRR and online supplemental Table A4 for crude IRR).

**Table 1:** Incidence rate (IR) per 100 000 person-years and adjusted incidence rate ratios (IRR) for hip arthroplasty due to OA for the selected biomechanical exposure factors

Job exposure matrix	Hip cohort					
	N	Person-years	Cases	IR	IRR*	95% CI
Time spent kneeling						
References†	10 803	253 491	423	167	1.00	Ref
Low	69 189	1 567 317	2871	183	1.05	0.95 to 1.17
Moderate	182 624	3 785 470	6038	160	0.95	0.86 to 1.05
High	28 435	620 442	1004	162	0.98	0.88 to 1.10
Frequency of heavy lifting up to 25 kg						
References†	10 803	253 491	423	167	1.00	Ref
Low	82 145	1 873 307	2984	159	1.01	0.91 to 1.11
Moderate	196 884	4 071 416	6879	169	0.97	0.88 to 1.07
High	1219	28 506	50	175	1.18	0.88 to 1.58
Heavy loading of the back – not necessarily related to lifting						
References†	10 803	253 491	423	167	1.00	Ref
Low	41 262	972 195	1787	184	1.11	1.00 to 1.24
Moderate	189 834	4 054 780	6310	156	0.97	0.88 to 1.07
High	49 152	946 253	1816	192	0.92	0.83 to 1.02
Frequency of repetitive lifting >25 kg						
References†	10 803	253 491	423	167	1.00	Ref
Low	105 237	2 355 555	3635	154	0.98	0.89 to 1.08
Moderate	59 898	1 293 318	2124	164	0.95	0.85 to 1.05
High	115 113	2 324 355	4154	179	1.00	0.91 to 1.11
Frequency of extreme trunk postures						
References†	10 803	253 491	423	167	1.00	Ref
Low	49 455	1 158 553	2166	187	1.11	1.00 to 1.24
Moderate	156 462	3 314 277	5065	153	0.95	0.86 to 1.05
High	74 331	1 500 398	2682	179	0.95	0.85 to 1.05
Frequency of static work in non-neutral lumbar postures						
References†	10 803	253 491	423	167	1.00	Ref
Low	51 999	1 215 470	2235	184	1.10	1.00 to 1.23
Moderate	221 943	4 618 534	7449	161	0.95	0.86 to 1.04
High	6306	139 224	229	165	1.09	0.93 to 1.28
Frequency of climbing stairs or ladders						
References†	10 803	253 491	423	167	1.00	Ref
Low	67 176	1 531 108	2788	182	1.06	0.96 to 1.18
Moderate	141 755	2 924 969	5061	173	0.99	0.89 to 1.09
High	71 317	1 517 152	2064	136	0.87	0.79 to 0.97
Magnitude of whole-body vibration						
References†	10 803	253 491	423	167	1.00	Ref
Low	250 361	5 307 283	8655	163	0.97	0.88 to 1.08
Moderate	15 783	328 435	627	191	0.97	0.86 to 1.10
High	14 104	337 510	631	187	1.10	0.97 to 1.24

* Analyses were adjusted for smoking habits, body mass index and time-varying age and calendar year throughout the follow-up period.

† White-collar workers were used as references.

N, number of workers; OA, osteoarthritis.

**Table 2:** Incidence rate (IR) per 100 000 person-years and adjusted incidence rate ratios (IRR) for knee arthroplasty due to OA, for the selected biomechanical exposure factors

Job exposure matrix	Knee cohort					
	N	Person-years	Cases	IR	IRR*	95% CI
Time spent kneeling						
References†	10 803	255 105	289	113	1.00	Ref
Low	69 189	1 575 801	2188	139	1.11	0.98 to 1.26
Moderate	182 634	3 797 837	5489	145	1.20	1.06 to 1.35
High	28 436	622 010	960	154	1.27	1.12 to 1.45
Frequency of heavy lifting up to 25 kg						
References†	10 803	255 105	289	113	1.00	Ref
Low	82 146	1 881 564	2398	127	1.10	0.98 to 1.25
Moderate	196 894	4 085 427	6195	152	1.21	1.08 to 1.36
High	1219	28 656	44	154	1.35	0.99 to 1.86
Heavy loading of the back – not necessarily related to lifting						
References†	10 803	255 105	289	113	1.00	Ref
Low	41 262	977 787	1326	136	1.15	1.01 to 1.31
Moderate	189 843	4 066 920	5828	143	1.22	1.08 to 1.37
High	49 154	950 940	1483	156	1.08	0.95 to 1.22
Frequency of repetitive lifting >25 kg						
References†	10 803	255 105	289	113	1.00	Ref
Low	105 240	2 364 516	3090	131	1.14	1.01 to 1.28
Moderate	59 900	1 297 541	1856	143	1.12	0.99 to 1.27
High	115 119	2 333 591	3691	158	1.25	1.11 to 1.41
Frequency of extreme trunk postures						
References†	10 803	255 105	289	113	1.00	Ref
Low	49 455	1 165 374	1622	139	1.15	1.02 to 1.31
Moderate	156 470	3 324 172	4660	140	1.20	1.06 to 1.35
High	74 334	1 506 101	2355	156	1.17	1.03 to 1.32
Frequency of static work in non-neutral lumbar postures						
References†	10 803	255 105	289	113	1.00	Ref
Low	51 999	1 222 507	1678	137	1.14	1.01 to 1.30
Moderate	221 954	4 633 344	6745	146	1.18	1.05 to 1.33
High	6306	139 796	214	153	1.38	1.16 to 1.65
Frequency of climbing stairs or ladders						
References†	10 803	255 105	289	113	1.00	Ref
Low	67 176	1 539 171	2138	139	1.12	0.99 to 1.27
Moderate	141 763	2 935 762	4497	153	1.22	1.08 to 1.37
High	71 320	1 520 714	2002	132	1.16	1.02 to 1.31
Magnitude of whole-body vibration						
References†	10 803	255 105	289	113	1.00	Ref
None	250 372	5 326 131	7688	144	1.20	1.06 to 1.35
Acceptable	15 783	329 960	503	152	1.08	0.93 to 1.25
High	14 104	339 556	446	131	1.02	0.88 to 1.18

* Analyses were adjusted for smoking habits, body mass index and time-varying age and calendar year throughout the follow-up period.

† White-collar workers were used as references.

N, number of workers; OA, osteoarthritis.

### Associations with occupational groups

Within the hip cohort, three of the 21 occupational groups; asphalt workers, crane operators and foremen showed an increased risk of hip arthroplasty compared with white-collar workers (IRR range 1.13 to 1.23). Conversely, four occupational groups: refrigerators, insulators, electricians and painters demonstrated a decreased risk (IRR range 0.60 to 0.84) (online supplemental Table A2). In the knee cohort, eight of the 21 occupational groups exhibited an increased risk of knee arthroplasty compared with white-collar workers. The highest risk was observed among floor layers, brick layers and wood workers, with IRR ranging from 1.36 to 1.39 (online supplemental Table A3).

### Self-reported biomechanical exposure

The hip subgroup that responded to the questionnaire on self-reported biomechanical exposure to working postures, including heavy lifting, non-neutral postures and kneeling, consisted of 70 231 individuals, while the knee subgroup included 70 251 individuals, representing approximately 24% of the total study cohort. In the analysis of the hip subgroup, only limited associations with an increased risk of future hip arthroplasty were found. The largest IRR reported was 1.22 (95% CI 1.02 to 1.45) (see table 3 for adjusted IRR and online supplemental Table A5 for crude IRR).

**Table 3:** Incidence rate (IR) per 100 000 person-years and adjusted incidence rate ratios (IRR) for hip arthroplasty due to OA, for self-reported biomechanical exposure

Self-reported biomechanical exposure	Hip cohort					
	N	Person-years	Cases	IR	IRR*	95% CI
Self-reported frequency of heavy lifting						
Rarely	9428	215 280	385	179	1.00	Ref
Quite rarely	5590	115 636	187	162	0.98	0.82 to 1.17
Sometimes	23 470	477 541	780	163	1.07	0.94 to 1.21
Quite often	18 670	378 371	633	167	1.17	1.03 to 1.32
Often	11 611	239 577	402	168	1.17	1.01 to 1.34
Self-reported frequency of working in non-neutral working postures						
Rarely	8491	193 110	348	180	1.00	Ref
Quite rarely	5332	107 436	205	191	1.22	1.02 to 1.45
Sometimes	17 768	357 570	573	160	0.98	0.86 to 1.12
Quite often	19 816	405 222	652	161	1.04	0.91 to 1.18
Often	17 354	363 114	600	165	1.04	0.91 to 1.18
Self-reported frequency of working in kneeling positions						
Rarely	10 248	235 218	435	185	1.00	Ref
Quite rarely	4155	87 552	162	185	1.04	0.87 to 1.24
Sometimes	14 830	303 456	494	163	0.95	0.84 to 1.08
Quite often	19 633	398 465	654	164	1.08	0.96 to 1.22
Often	19 952	402 725	639	159	1.00	0.89 to 1.13

* Analyses were adjusted for smoking habits, body mass index and time-varying age and calendar year throughout the follow-up period.

N, number of workers; OA, osteoarthritis.

In the knee subgroup, individuals who reported frequent heavy lifting had a slightly elevated risk of knee arthroplasty compared with those with infrequent exposure, with an IRR of 1.30 (95% CI 1.14 to 1.50). Similar, though smaller, increases in risk were observed for non-neutral working postures and kneeling. However, no clear exposure-response patterns were identified for any of the three self-reported exposures (see table 4 for adjusted IRR and online supplemental Table A5 for crude IRR).

**Table 4:** Incidence rate (IR) per 100 000 person-years and adjusted incidence rate ratios (RR) for knee arthroplasty due to OA, for self-reported biomechanical exposure

Self-reported biomechanical exposure	Knee cohort					
	N	Person-years	Cases	IR	IRR*	95% CI
Self-reported frequency of heavy lifting						
Rarely	9433	216 248	327	151	1.00	Ref
Quite rarely	5591	116 057	171	147	1.06	0.88 to 1.28
Sometimes	23 476	479 718	640	133	1.03	0.90 to 1.18
Quite often	18 674	379 868	589	155	1.30	1.14 to 1.50
Often	11 612	240 548	360	150	1.22	1.05 to 1.42
Self-reported frequency of working in non-neutral working postures						
Rarely	8494	194 195	293	151	1.00	Ref
Quite rarely	5333	108 191	135	125	0.96	0.79 to 1.18
Sometimes	17 776	359 186	480	134	0.97	0.84 to 1.13
Quite often	19 819	406 402	629	155	1.16	1.01 to 1.34
Often	17 356	364 498	546	150	1.10	0.95 to 1.26
Self-reported frequency of working in kneeling positions						
Rarely	10 251	236 590	345	146	1.00	Ref
Quite rarely	4156	87 989	143	163	1.18	0.97 to 1.43
Sometimes	14 834	304 691	409	134	0.99	0.86 to 1.14
Quite often	19 636	399 848	596	149	1.24	1.08 to 1.42
Often	19 958	404 306	598	148	1.19	1.04 to 1.36

* Analyses were adjusted for smoking habits, body mass index and time-varying age and calendar year throughout the follow-up period.

N, number of workers; OA, osteoarthritis.

## Discussion

This study aimed to enhance our understanding of the relationship between occupational biomechanical exposures and the need for surgical treatment of OA with arthroplasty among male construction workers. Using the national Construction Workers Cohort, previously used in a similar study by Järvholm *et al*,^15^ we extended the follow-up period from 12 to 33 years and improved our exposure assessment by incorporating a JEM alongside subjective ratings.

Our findings are in concordance with those of Järvholm *et al*,^15^ reinforcing the association between occupational groups and an increased risk of severe knee OA requiring arthroplasty. However, the association between occupational groups and hip OA remains less clear. We observed the highest increased risk of knee arthroplasty among floor layers, bricklayers and woodworkers, with IRRs ranging from 1.36 to 1.39 compared with white-collar workers. This partially aligns with the earlier study, where the highest risks were reported among floor layers, asphalt workers, sheet-metal workers and rock workers.^15^ For hip arthroplasty, we identified modestly increased risks among asphalt workers (IRR 1.23), crane operators (IRR 1.22) and foremen (IRR 1.13). In contrast, Järvholm *et al* did not find significant associations with hip OA, possibly due to a shorter follow-up time.^15^

We also examined biomechanical risk factors using a JEM. Our findings support the association between these exposures and knee OA, with most of the examined biomechanical factors demonstrating exposure-response patterns. Although the IRRs and their upper CIs limits were modest, they consistently indicated an increased risk, and even small relative increases may have an impact in large occupational groups with long-term and repeated exposures to heavy biomechanical loads. The highest risks were linked to static working in non-neutral lumbar postures, frequent heavy lifting and frequent kneeling (IRR ranging from 1.27 to 1.38). These activities are commonly performed by floor layers, brick layers, wood workers and roofers. Additionally, analysis of self-reported exposure ratings reinforced these associations, showing an increased risk of knee arthroplasty in individuals reporting frequent heavy lifting, non-neutral postures and kneeling (IRR ranging from 1.16 to 1.30). However, it should be noted that individuals with existing knee symptoms might overestimate their exposure to activities that strain the knees.^22^ Our results are consistent with prior research, including a systematic review by Wang *et al*^11^ that identified multiple associations with exposure factors such as climbing, squatting and lifting, with pooled OR ranging from 1.39 to 1.49. The highest probability of knee OA was observed in floor and brick layers (OR 2.51 (95% CI 1.79 to 3.52)) and carpenters (OR 2.49 (95% CI 1.66 to 3.74)). Similarly, Canetti *et al*^9^ reported that frequent squatting or kneeling (OR 1.69 (95% CI 1.15 to 2.49)) and heavy lifting (OR 1.52 (95% CI 1.29 to 1.79)) significantly increased the probability of knee OA.

Furthermore, the study investigated the relationship between biomechanical exposures and hip OA, but found no significant associations between the measured factors from the JEM and hip arthroplasty. A modest increase in the risk was observed among individuals who were exposed to frequently heavy lifting (IRR 1.17) compared with those who rarely did so. The associations were not only statistically non-significant, but the relatively low upper bounds of the CIs suggest that any potential associations are likely to be weak. In contrast, previous reviews have shown significant associations between occupational exposures and hip OA,^9 14^ with Canetti *et al*^9^ reporting an OR of 1.35 (95% CI 1.16 to 1.57) for heavy lifting and Jahn *et al*^13^ noting associations with non-neutral postures (pooled OR 1.7 (95% CI 1.4 to 2.1)) and lifting/carrying (pooled OR 1.6 (95% CI 1.3 to 1.9)).

The differences in study populations, occupational types, study designs and, most notably, definitions of OA make comparisons complicated.^14^ Some studies define OA through clinical symptoms, radiographic findings or ICD-10 codes, while others, like ours, use treatment with arthroplasty as a measure of severe OA.^9 11 13^ Overall, a high physical workload has consistently been linked to a higher risk of knee OA, but the evidence for hip OA is less conclusive.^15 23 24^

This distinction between hip and knee OA identified in this study, as well as in prior research,^15 24^ is noteworthy. However, this difference may be explained by substantial differences between the conditions.^25^ For example, genetic factors and joint deformities are likely to have a greater influence on the development of hip OA, while knee OA is more significantly affected by biomechanics and joint load.^25^,^27^ This understanding aligns with our findings, which identify associations between occupational biomechanical exposures and knee arthroplasty but show limited associations with hip arthroplasty. Given the large number of cases with hip arthroplasty, insufficient statistical power is unlikely to explain the lack of associations with biomechanical exposures. Instead, this consistent absence across exposure measures suggests possible aetiological differences, with occupational biomechanical load appearing more relevant for knee OA than for hip OA. In a previous study from the Construction Workers Cohort, with follow-up time until 1998 there were no statistically significant risks for hip arthroplasty in any of the occupational groups.^15^

Some methodological considerations should be addressed. Hip and knee OA are distinct conditions with different causes, risk factors, clinical presentation,^25^ surgical rates, results and timing of surgery.^28^ Therefore, we divided our cohort into a hip and knee subgroup, contrasting the study by Järvholm *et al*.^15^ In our study, individuals remained at risk of knee surgery even after undergoing hip surgery during the follow-up period, and vice versa. This overlap limits direct comparisons of IRR between the two studies. We also acknowledge that there has been a general increase in surgeries over time.^29 30^ In this study, we report an IR of 166 hip arthroplasties and 143 knee arthroplasties per 100 000 person-years from 1987 to 2019. Data from the National Board of Health and Welfare indicate that hip arthroplasties rose from 178 per 100 000 residents in 1998 to 286 in 2019, and knee arthroplasties increased from 96 to 261 surgeries during the same period.^31^ This rising trend of procedures aligns with global patterns and is expected to continue.^29 30 32^ To account for this, we used a time-split analysis, which enabled us to separate the time scales of age and calendar year and adjust for both.

Furthermore, data on job title, BMI and smoking habits were collected during health examinations and not updated during follow-up. This may have resulted in exposure misclassification and required the assumption that workers remained in the same occupational group and that BMI and smoking habits stayed relatively stable over time. Although personal factors such as BMI and smoking may change, longitudinal studies show substantial tracking of BMI across adulthood and persistence in smoking behaviour.^33 34^ A previous study from Sweden supported this assumption, as construction workers typically stayed within the same occupational group,^19^ suggesting that changes in biomechanical exposure due to job mobility are limited. Any resulting misclassification is likely non-differential and would bias the results toward the null.

It is also essential to recognise that the process leading to arthroplasty is multifactorial, incorporating individual willingness to undergo surgery and socioeconomic factors,^28^ access to healthcare and surgeons’ decision-making. These factors may affect the likelihood of receiving arthroplasty and therefore represent a potential source of outcome misclassification and bias.

We used a cohort-specific JEM, developed through a blind procedure where two ergonomic expert researchers independently rated the average intensity or frequency of exposure throughout a working day for each job title. This assessment was based on ergonomic evaluations conducted in the 1970s. While this method has limitations, particularly the potential for exposure misclassification due to the absence of direct and quantified evaluations of biomechanical exposure, it remains the best approach for assessing exposure in large-scale epidemiological studies.^35^ In a subgroup of the cohort, we also analysed self-reported exposure data. Self-reported information may introduce bias, for example, if individuals overestimate or underestimate exposures in relation to symptoms or perceived risk.^22^ However, the consistency between results obtained from the self-reported data and the JEM-based exposure assessments for both hip and knee arthroplasty strengthens the validity of our findings and suggests that self-reporting bias is unlikely to have materially influenced the overall conclusions. Lastly, our study included only male construction workers, even though the proportion of women in construction trades steadily increases.

To the best of our knowledge, this is the largest study conducted to date on biomechanical risk factors for hip and knee arthroplasty. It is based on a large national cohort that is highly representative of Swedish male construction workers and covers an extended follow-up period, which strengthens the validity of the findings. The results are confirmatory in nature and support existing evidence on biomechanical exposures and OA-related arthroplasty. These findings have important public health implications, particularly for developing targeted prevention strategies in the construction industry.

In conclusion, our findings confirm and extend existing evidence that occupational workload is associated with an increased risk of knee arthroplasty due to OA. This emphasises the need to reduce exposure to specific occupational activities, such as static work in non-neutral lumbar postures, heavy lifting and kneeling, to prevent the development of knee OA among workers in the construction industry. In contrast, we observed no clear associations with hip arthroplasty, indicating that occupational prevention strategies for hip OA remain less well understood and warrant further investigation.

## Supplementary material

10.1136/bmjopen-2025-107604 online supplemental file 1

## Data Availability

Data are available on reasonable request.
