# Supplementary material for: Occupational biomechanical risk factors for hip and knee arthroplasty incidence: a register-based cohort study in male construction workers
Source: BMJ Open. 2026 Apr 6;16(4):e107604. doi: 10.1136/bmjopen-2025-107604 (PMC13055328; doi:10.1136/bmjopen-2025-107604)
Supplement: online supplemental file 1 [file bmjopen-16-4-s001.docx]

# **Supplementary material**

**Table A1.** Description of the occupational groups and their ratings for the factors included from the Job Exposure Matrix (JEM).

| **Description of JEM factors** | **Occupational group** | | | | | | | | | | | | | | | | | | | | |
| --- | --- | --- | --- | --- | --- | --- | --- | --- | --- | --- | --- | --- | --- | --- | --- | --- | --- | --- | --- | --- | --- |
|  | Asphalt workers | Rock workers | Concrete workers | Wood workers | Brick layers | Floor layers | Heavy machinery operators operators | Crane operators | Drivers | Glass workers | Insulators | Refrigerator technicians | Plumbers | Painters | Sheet-metal workers | Electricians | Foremen | White-collar workers | Repairers | Preparatory workers | Roofers |
| Time spent kneeling | 1 | 1 | 2 | 2 | 2 | 3 | 1 | 1 | 1 | 1 | 1 | 2 | 3 | 2 | 2 | 2 | 1 | 1 | 2 | 1 | 2 |
| Frequency of heavy lifting up to 25 kg | 1 | 2 | 2 | 2 | 2 | 2 | 1 | 1 | 2 | 2 | 1 | 2 | 2 | 2 | 2 | 1 | 1 | 1 | 2 | 2 | 3 |
| Heavy loading of the back – not necessarily related to lifting | 2 | 3 | 3 | 2 | 3 | 2 | 1 | 1 | 2 | 2 | 2 | 2 | 2 | 2 | 2 | 2 | 1 | 1 | 2 | 2 | 3 |
| Frequency of repetitive lifting >25 kg | 1 | 2 | 3 | 3 | 3 | 3 | 1 | 1 | 2 | 2 | 1 | 2 | 2 | 1 | 2 | 1 | 1 | 1 | 2 | 2 | 3 |
| Frequency of extreme trunk postures | 1 | 2 | 3 | 2 | 3 | 3 | 1 | 1 | 1 | 2 | 2 | 2 | 3 | 2 | 2 | 2 | 1 | 1 | 2 | 2 | 3 |
| Frequency of static work in non-neutral lumbar postures | 1 | 2 | 2 | 2 | 2 | 3 | 1 | 1 | 1 | 1 | 2 | 2 | 2 | 2 | 2 | 2 | 1 | 1 | 2 | 2 | 3 |
| Frequency of climbing stairs or ladders | 1 | 2 | 2 | 2 | 3 | 2 | 1 | 1 | 1 | 1 | 3 | 1 | 2 | 3 | 2 | 3 | 1 | 1 | 1 | 1 | 3 |
| Magnitude of whole body vibration* | 3 | 1 | 1 | 1 | 1 | 1 | 3 | 1 | 2 | 1 | 1 | 1 | 1 | 1 | 1 | 1 | 1 | 1 | 1 | 2 | 1 |

1=low, 2=moderate, 3=high

* 1=None, 2= acceptable, 3= high

**Table A2**. Incidence rates (IR) per 100 000 person-years and incidence rate ratios (IRR) for hip arthroplasty, according to occupational group. Occupational groups shown in order of decreasing adjusted IRR.

|  | **Hip cohort** | | | | | |
| --- | --- | --- | --- | --- | --- | --- |
| **Occupational groups** | **N** | **Person-years** | **Cases** | **IR** | **IRR**^a^  **(95% CI)** | **IRR^b^**  **(95% CI)** |
| White-collar workers | 10 803 | 253 491 | 423 | 167 | Reference | Reference |
| Asphalt workers | 3791 | 87 678 | 192 | 219 | 1.31  (1.11 to 1.56) | 1.23  (1.04 to 1.46) |
| Crane operators | 3164 | 76 548 | 161 | 210 | 1.26  (1.05 to 1.51) | 1.22  (1.01 to 1.46) |
| Roofers | 1219 | 28 506 | 50 | 175 | 1.05  (0.78 to 1.41) | 1.18  (0.88 to 1.58) |
| Foremen | 27 785 | 645 815 | 1187 | 184 | 1.10  (0.99 to 1.23) | 1.13  (1.01 to 1.26) |
| Floor layers | 5087 | 110 718 | 179 | 162 | 0.97  (0.81 to 1.15) | 1.06  (0.89 to 1.27) |
| Wood workers | 64 130 | 1 333 680 | 2297 | 172 | 1.03  (0.93 to 1.14) | 1.06  (0.95 to 1.17) |
| Heavy machinery operators | 10 313 | 249 832 | 439 | 176 | 1.05  (0.92 to 1.20) | 1.04  (0.91 to 1.19) |
| Drivers | 4402 | 98 680 | 187 | 190 | 1.14  (0.96 to 1.35) | 1.00  (0.85 to 1.19) |
| Brick layers | 9914 | 192 963 | 358 | 186 | 1.11  (0.97 to 1.28) | 0.99  (0.86 to 1.14) |
| Plumbers | 23 348 | 509 724 | 825 | 162 | 0.97  (0.86 to 1.09) | 0.97  (0.86 to 1.09) |
| Sheet-metal workers | 11 171 | 246 064 | 352 | 143 | 0.86  (0.74 to 0.99) | 0.97  (0.85 to 1.12) |
| Preparatory workers | 11 381 | 229 755 | 440 | 192 | 1.15  (1.00 to 1.31) | 0.96  (0.84 to 1.10) |
| Concrete workers | 34 763 | 658 488 | 1270 | 193 | 1.16  (1.04 to 1.29) | 0.90  (0.80 to 1.00) |
| Painters | 23 092 | 482 249 | 651 | 135 | 0.81  (0.72 to 0.91) | 0.87  (0.77 to 0.99) |
| Glass workers | 2544 | 56 918 | 69 | 121 | 0.73  (0.56 to 0.94) | 0.87  (0.67 to 1.12) |
| Rock workers | 3256 | 66 296 | 138 | 208 | 1.25  (1.03 to 1.51) | 0.87  (0.72 to 1.05) |
| Repairers | 2595 | 59 123 | 89 | 151 | 0.90  (0.72 to 1.13) | 0.86  (0.68 to 1.08) |
| Electricians | 34 539 | 757 639 | 947 | 125 | 0.75  (0.67 to 0.84) | 0.84  (0.75 to 0.95) |
| Insulators | 2553 | 55 795 | 58 | 104 | 0.62  (0.47 to 0.82) | 0.66  (0.50 to 0.87) |
| Refrigerator technicians | 1201 | 26 759 | 24 | 90 | 0.54  (0.36 to 0.81) | 0.60  (0.40 to 0.91) |

N, number of workers; IR, Incidence rate; IRR, incidence risk ratio; CI, confidence interval.

^a^ Crude analyses.

^b^ Analyses were adjusted for smoking habits, body mass index (BMI), and time-varying age and calendar year throughout the follow-up period.

**Table A3.** Incidence rate (IR) per 100 000 person-years and incidence rate ratios (IRR) for knee arthroplasty, according to occupational group. Occupational groups shown in order of decreasing adjusted IRR.

|  | **Knee cohort** | | | | | |
| --- | --- | --- | --- | --- | --- | --- |
| **Occupational groups** | **N** | **Person-years** | **Cases** | **IR** | **IRR**^a^  **(95% CI)** | **IRR^b^**  **(95% CI)** |
| White-collar workers | 10 803 | 255 105 | 298 | 113 | Reference | Reference |
| Floor layers | 5087 | 111 141 | 170 | 153 | 1.35  (1.12 to 1.63) | 1.39  (1.15 to 1.68) |
| Brick layers | 9914 | 193 592 | 341 | 176 | 1.55  (1.33 to 1.82) | 1.37  (1.17 to 1.60) |
| Wood workers | 64 134 | 1 338 359 | 2126 | 159 | 1.40  (1.24 to 1.59) | 1.36  (1.20 to 1.54) |
| Roofers | 1219 | 28 656 | 44 | 154 | 1.36  (0.99 to 1.86) | 1.36  (0.99 to 1.86) |
| Painters | 23 094 | 482 952 | 692 | 143 | 1.26  (1.10 to 1.45) | 1.27  (1.11 to 1.46) |
| Asphalt workers | 3791 | 88 263 | 147 | 167 | 1.47  (1.21 to 1.79) | 1.27  (1.04 to 1.55) |
| Plumbers | 23 349 | 510 869 | 790 | 155 | 1.36  (1.19 to 1.56) | 1.25  (1.09 to 1.43) |
| Crane operators | 3164 | 77 079 | 118 | 153 | 1.35  (1.09 to 1.67) | 1.25  (1.01 to 1.55) |
| Foremen | 27 785 | 649 415 | 909 | 140 | 1.24  (1.08 to 1.41) | 1.23  (1.08 to 1.41) |
| Sheet-metal workers | 11 172 | 246 702 | 313 | 127 | 1.12  (0.95 to 1.31) | 1.15  (0.98 to 1.35) |
| Refrigerator technicians | 1201 | 26 716 | 35 | 131 | 1.16  (0.81 to 1.64) | 1.11  (0.78 to 1.58) |
| Preparatory workers | 11 381 | 230 636 | 354 | 154 | 1.35  (1.16 to 1.58) | 1.08  (0.92 to 1.26) |
| Drivers | 4402 | 99 324 | 149 | 150 | 1.32  (1.09 to 1.61) | 1.07  (0.88 to 1.31) |
| Insulators | 2553 | 55 809 | 68 | 122 | 1.08  (0.83 to 1.40) | 1.06  (0.81 to 1.38) |
| Electricians | 34 540 | 759 706 | 857 | 113 | 1.00  (0.87 to 1.14) | 1.03  (0.90 to 1.18) |
| Concrete workers | 34 765 | 661 844 | 1010 | 153 | 1.35  (1.18 to 1.54) | 1.02  (0.90 to 1.17) |
| Heavy machinery operators | 10 313 | 251 293 | 299 | 119 | 1.05  (0.89 to 1.23) | 0.93  (0.79 to 1.09) |
| Repairers | 2595 | 59 312 | 71 | 120 | 1.06  (0.82 to 1.37) | 0.92  (0.71 to 1.20) |
| Glass workers | 2544 | 57 133 | 56 | 98 | 0.87  (0.65 to 1.15) | 0.92  (0.69 to 1.22) |
| Rock workers | 3256 | 66 849 | 88 | 132 | 1.16  (0.92 to 1.48) | 0.80  (0.63 to 1.01) |

N, number of workers; IR, Incidence rate; IRR, incidence risk ratio; CI, confidence interval.

^a^ Crude analyses.

^b^ Analyses were adjusted for smoking habits, body mass index (BMI), and time-varying age and calendar year throughout the follow-up period.

**Table A4.** Crude incidence risk ratios (IRR) for hip and knee arthroplasty due to OA for the selected biomechanical exposure factors.

|  | **Hip cohort** | | **Knee cohort** | |
| --- | --- | --- | --- | --- |
| **Job exposure matrix (JEM)** | **IRR**^b^ | **95% CI** | **IRR**^b^ | **95% CI** |
|  | **Time spent kneeling** | | | |
| References^a^ | 1.00 | Ref | 1.00 | Ref |
| Low | 1.10 | 0.99 to 1.22 | 1.23 | 1.08 to 1.39 |
| Moderate | 0.96 | 0.87 to 1.05 | 1.28 | 1.13 to 1.44 |
| High | 0.97 | 0.87 to 1.09 | 1.36 | 1.19 to 1.55 |
|  | **Frequency of heavy lifting up to 25 kg** | | | |
| References^a^ | 1.00 | Ref | 1.00 | Ref |
| Low | 0.95 | 0.86 to 1.06 | 1.12 | 1.00 to 1.27 |
| Moderate | 1.01 | 0.92 to 1.12 | 1.34 | 1.19 to 1.51 |
| High | 1.05 | 0.78 to 1.41 | 1.36 | 0.99 to 1.86 |
|  | **Heavy loading of the back – not necessarily related to lifting** | | | |
| References^a^ | 1.00 | Ref | 1.00 | Ref |
| Low | 1.10 | 0.99 to 1.22 | 1.20 | 1.05 to 1.36 |
| Moderate | 0.93 | 0.85 to 1.03 | 1.26 | 1.12 to 1.42 |
| High | 1.15 | 1.03 to 1.28 | 1.38 | 1.21 to 1.56 |
|  | **Frequency of repetitive lifting >25 kg** | | | |
| References^a^ | 1.00 | Ref | 1.00 | Ref |
| Low | 0.92 | 0.84 to 1.02 | 1.15 | 1.02 to 1.30 |
| Moderate | 0.98 | 0.89 to 1.09 | 1.26 | 1.12 to 1.43 |
| High | 1.07 | 0.97 to 1.18 | 1.40 | 1.24 to 1.57 |
|  | **Frequency of extreme trunk postures** | | | |
| References^a^ | 1.00 | Ref | 1.00 | Ref |
| Low | 1.12 | 1.01 to 1.24 | 1.23 | 1.08 to 1.39 |
| Moderate | 0.92 | 0.83 to 1.01 | 1.24 | 1.10 to 1.39 |
| High | 1.07 | 0.97 to 1.19 | 1.38 | 1.22 to 1.56 |
|  | **Frequency of static work in non-neutral lumbar postures** | | | |
| References^a^ | 1.00 | Ref | 1.00 | Ref |
| Low | 1.10 | 0.99 to 1.22 | 1.21 | 1.07 to 1.37 |
| Moderate | 0.97 | 0.88 to 1.07 | 1.28 | 1.14 to 1.45 |
| High | 0.99 | 0.84 to 1.16 | 1.35 | 1.13 to 1.61 |
|  | **Frequency of climbing stairs or ladders** | | | |
| References^a^ | 1.00 | Ref | 1.00 | Ref |
| Low | 1.09 | 0.99 to 1.21 | 1.23 | 1.08 to 1.39 |
| Moderate | 1.04 | 0.94 to 1.14 | 1.35 | 1.20 to 1.52 |
| High | 0.82 | 0.73 to 0.91 | 1.16 | 1.03 to 1.31 |
|  | **Magnitude of whole-body vibration** | | | |
| References^a^ | 1.00 | Ref | 1.00 | Ref |
| None | 0.98 | 0.89 to 1.08 | 1.27 | 1.13 to 1.43 |
| Acceptable | 1.14 | 1.01 to 1.29 | 1.35 | 1.16 to 1.56 |
| High | 1.12 | 0.99 to 1.27 | 1.16 | 1.00 to 1.34 |

IRR, incidence risk ratio; CI, confidence interval.

^a^ White-collar workers were used as references

^b^ Crude analyses.

**Table A5.** Crude incidence risk ratios (IRR) for hip and knee arthroplasty due to OA, for self-reported biomechanical exposure.

|  | **Hip cohort** | | **Knee cohort** | |
| --- | --- | --- | --- | --- |
| **Self-reported biomechanical exposure** | **IRR**^a^ | **95% CI** | **IRR**^a^ | **95% CI** |
|  | **Self-reported frequency of heavy lifting** | | | |
| Rarely | 1.00 | Ref | 1.00 | Ref |
| Quite rarely | 0.90 | 0.76 to 1.08 | 0.97 | 0.81 to 1.17 |
| Sometimes | 0.91 | 0.81 to 1.03 | 0.88 | 0.77 to 1.01 |
| Quite often | 0.94 | 0.82 to 1.06 | 1.03 | 0.90 to 1.17 |
| Often | 0.94 | 0.82 to 1.08 | 0.99 | 0.85 to 1.15 |
|  | **Self-reported frequency of working in non-neutral working postures** | | | |
| Rarely | 1.00 | Ref | 1.00 | Ref |
| Quite rarely | 1.06 | 0.89 to 1.26 | 0.83 | 0.67 to 1.01 |
| Sometimes | 0.89 | 0.78 to 1.02 | 0.89 | 0.77 to 1.02 |
| Quite often | 0.89 | 0.78 to 1.02 | 1.03 | 0.89 to 1.18 |
| Often | 0.92 | 0.80 to 1.05 | 0.99 | 0.86 to 1.14 |
|  | **Self-reported frequency of working in kneeling positions** | | | |
| Rarely | 1.00 | Ref | 1.00 | Ref |
| Quite rarely | 1.00 | 0.84 to 1.20 | 1.11 | 0.92 to 1.35 |
| Sometimes | 0.88 | 0.77 to 1.00 | 0.92 | 0.80 to 1.06 |
| Quite often | 0.89 | 0.79 to 1.00 | 1.02 | 0.90 to 1.17 |
| Often | 0.86 | 0.76 to 0.97 | 1.01 | 0.89 to 1.16 |

IRR, incidence risk ratio; CI, confidence interval.

^a^ Crude analyses

Excluded (in total; n = 4149)

- Female (n = 3405)
- Aged <15 or >67 years at enrollment (n = 41)
- Missing data height or weight (n =549)
- Height <150 cm or >200 cm (n = 79)
- BMI <17 or >40 kg/m^2^ (n = 166)

Remaining cohort (n = 82 956)

**Hip cohort**

**(n = 70 231)**

Excluded

|  | Hip cohort | Knee cohort |
| --- | --- | --- |
| - Workers with job titles (trades) that could not be mapped onto the JEM | n = 10 803 | n = 10 807 |

Lost to follow-up

(in total; hip cohort: n = 1922, knee cohort: n = 1898)

|  | Hip cohort | Knee cohort |
| --- | --- | --- |
| - <40 years when becoming a case | n = 27 | n = 3 |
| - Died pre-observation period | n = 198 | n = 198 |
| - Emigrated pre-observation period | n = 1703 | n = 1703 |
| - Turning 80 pre-observation period | n = 0 | n = 0 |

## Follow-up

## Enrollment

## JEM

Total database of workers replying to at least one of the questionnaires regarding self-reported biomechanical exposure, between 1989 and 1993 (n = 87 105)

**Knee cohort**

**(n = 70 251)**

Remaining cohorts

(Hip: n = 81 034, knee: n = 81 058)

**Figure A1.** Flow chart of the subgroup responding to the questionnaire regarding self-reported biomechanical exposure.
