# Supplementary material for: Occupational biomechanical risk factors for hip and knee arthroplasty incidence: a register-based cohort study in male construction workers
Source: BMJ Open. 2026 Apr 6;16(4):e107604. doi: 10.1136/bmjopen-2025-107604 (PMC13055328; doi:10.1136/bmjopen-2025-107604)
Supplement: Supplementary file 2 [file bmjopen-2025-107604.reviewer_comments.pdf]

PEER REVIEW HISTORY

BMJ Open publishes all reviews undertaken for accepted manuscripts. Reviewers are asked to complete a checklist review form and are provided with free text boxes to elaborate on their assessment. These free text comments are reproduced below.

ARTICLE DETAILS

Title (Provisional)

Occupational biomechanical risk factors for hip and knee arthroplasty incidence: a register-based cohort study in male construction workers

Authors

Gustafsson, Kristin; Wahlström, Jens; Stjernbrandt, Albin; Lewis, Charlotte; Mukka, Sebastian; Liv, Per; Noor Baloch, Adnan

---

VERSION 1 - REVIEW

Reviewer 1

Name Kang, Mo-Yeol

Affiliation College of Medicine, The Catholic University of Korea, Department of Occupational and Environmental Medicine

Date 16-Aug-2025

COI None

This manuscript investigates the association between occupational biomechanical exposures and the incidence of hip and knee arthroplasty due to osteoarthritis (OA) among Swedish male construction workers. The study is noteworthy for its extensive cohort, long follow-up duration, and use of both job-exposure matrix (JEM) and self-reported exposure data. While the findings reinforce previously established associations, the study's novelty is somewhat limited. The contribution lies primarily in its confirmatory strength rather than in generating new hypotheses or theoretical advancements. The manuscript is well-designed and executed. Minor revisions to clarify interpretations, emphasize contribution, and improve presentation will further strengthen its impact.

1. The conclusions largely confirm existing literature on biomechanical risk factors for knee OA. The authors are encouraged to emphasize the confirmatory and public health implications more explicitly in the discussion.

2. The null findings for hip OA could benefit from clearer interpretation. It should be specified whether this is due to insufficient statistical power or reflects true etiological differences.

3. Occupational titles, BMI, and smoking status were captured only at baseline. The implications of assuming stable exposure over decades should be addressed more thoroughly in the limitations.

4. The manuscript states that Poisson regression was used to estimate relative risks (RR). To avoid confusion with prevalence ratios, especially among general readers, the authors may consider clarifying that the model estimates incidence rate ratios (IRR) based on person-time data in a longitudinal cohort.

**Reviewer**	**2**
**Name**	**OZTERMELI, Ahmet**
**Affiliation**	**Gebze Fatih State Hospital, Ortopedics and Traumatology**
**Date**	**29-Dec-2025**
**COI**	**None**

Given the small magnitude of the observed risk increases, it is unclear whether these findings are clinically meaningful.

Occupational exposure, BMI, and smoking status were assessed only at the first health examination and were not updated during follow-up. This may have resulted in exposure misclassification and should be acknowledged more clearly as a limitation.

Arthroplasty reflects severe osteoarthritis but is also influenced by factors such as healthcare access, patient preferences, and surgical decision-making. These factors should be acknowledged as potential sources of bias.

**Reviewer**	**3**
**Name**	**Kelson, Zoe**
**Affiliation**	**University of Exeter, Mathematics**
**Date**	**03-Feb-2026**
**COI**	**None**

This study aims to evaluate the association between exposure to occupational biomechanical factors and the incidence of surgically osteoarthritis (OA) treated with arthroplasty in the hip and knee among male construction workers.

Reviewer comments:

"This longitudinal register-based cohort study followed male construction workers for 33 years to identify arthroplasties in the hip or knee due to OA and to explore the relationship between prior occupational biomechanical exposures, and the incidence of surgery"

The authors do well to utilise a large longitudinal dataset in this study.

"Male construction workers (n=291 062) who participated in a national Swedish occupational health examination program between 1971 and 1993 were followed from 1987 to 2019"

and

"Two outcome variables were utilized in this study: the first occurrence of surgically treated hip OA and knee OA, each assessed separately during the follow-up period from 1987 to 2019. "

Can the authors please consider whether follow-up data can be updated to more recent years?

"The study included 10 336 cases of hip arthroplasties and 8926 cases of knee arthroplasties" [Abstract]

and

"The study cohort was selected from a total of 389 132 Swedish construction workers who participated in health examinations as part of a national health surveillance program conducted from the late 1960s until 1993 (the Construction Workers Cohort). The construction workers were offered free, voluntary health checks through agreements established between employers and labor unions, with approximately 80% completing at least one health examination"

and

"In the study cohort, we included male construction workers examined between 1971 and 1993. We used data from each individual´s first health examination, including those aged 15 to 67."

Can the authors please further comment on whether the included cohort can be considered to be representative?

Can cohort demographics please be tabulated?

"Individuals with missing height or weight data, those who were unusually short (<150 cm) or tall (>200 cm), and those with a BMI outside the range of 17 to 40 kg/m 2 were excluded...

Furthermore, individuals not able to classify in a specific occupational group were excluded, since they could not be mapped according to the JEM (Figure 1)."

Can the authors please assess whether missing data can be considered to be missing at random?

"Data on age, smoking habits, body mass index (BMI), job title, and self-reported biomechanical exposures were collected during the health examinations. " [Abstract]

and

"Data on smoking habits (classified as never, ever, or unknown) and height and weight, from which BMI was caculated, were collected from each individual´s initial health examination. Age at the health examination was also recorded. "

Can the authors please confirm if there was any other missing data (e.g. age or BMI)?

If so, how was this missing data handled in the analysis?

"Each individual's job title was collected from their last registered health examination."

Can the authors please clarify how changes in job and occupation are handled in the analysis?

"Between 1989 and 1993, an additional questionnaire was used during the health examinations to gather data on self-reported exposure to various biomechanical exposures."

Can the authors please acknowledge the potential for self reporting bias, for these additional questions as well as other variables in the analysis?

"Poisson regression was applied to estimate adjusted relative risks (RR) associated with each type of occupational biomechanical exposure" [Abstract]

and

"Poisson regression analyses were conducted, designating biomechanical exposure as the primary independent variable and hip arthroplasty due to OA as the outcome variable...

Poisson regression models were utilized to calculate incidence rates (IR), wherein the counts of hip arthroplasty due to OA and person-years at risk were compared across levels of biomechanical exposures, including the logarithm of person-years at risk as offset...

All regression analyses were adjusted for age at follow-up and BMI at health examination (both modelled using restricted cubic splines with four knots positioned at the 5th, 35th, 65th, and 95th percentiles), smoking status, and the calendar year as a linear effect. Poisson regressions were performed for each of the biomechanical exposures derived from the JEM and for the self-reported biomechanical exposures (among the subgroup that responded to the questionnaire). All analyses were repeated using knee arthroplasty as the outcome"

Appropriate modelling methods have been applied by the authors.

Can the authors please expand on the description of the model, including how time-varying confounders were included as covariates and clarifying how the longitudinal data was analysed?

"The follow-up period began in the year after the initial health examination, on January 1, 1987, or in the year the individuals reached 40, whichever occurred later.

Observations were censored at instances of death, emigration, reaching the age of 80 years, or at the conclusion of the study period at December 31, 2019"

Can the authors please clarify if and how differences in follow-up time have been adjusted for in the analyses?

Did the authors please consider applying a Cox regression, given the time-to-event nature of the study?

"By including only male construction workers, the study´s findings may not be generalizable to women." [Strengths and Limitations]

and

"Some methodological considerations should be addressed..."

The authors provide a Discussion on the study limitations and assumptions.

Thanks for providing a copy of the STROBE checklist.

VERSION 1 - AUTHOR RESPONSE

Reviewer 1, specific comment, (Prof. Mo-Yeol Kang, College of Medicine, The Catholic University of Korea)
Comments to the Author:
This manuscript investigates the association between occupational biomechanical exposures and the incidence of hip and knee arthroplasty due to osteoarthritis (OA) among Swedish male construction workers. The study is noteworthy for its extensive cohort, long follow-up duration, and use of both job-exposure matrix (JEM) and self-reported exposure data. While the findings reinforce previously established associations, the study's novelty is somewhat limited. The contribution lies primarily in its confirmatory strength rather than in generating new hypotheses or theoretical advancements. The manuscript is well-designed and executed. Minor revisions to clarify interpretations, emphasize contribution, and improve presentation will further strengthen its impact.

Authors' response
Thank you for your positive feedback on our manuscript and for taking the time to provide insightful advice to further improve the clarity of our message. We respond to your comments point by point below.

Reviewer 1 Comment:
1. The conclusions largely confirm existing literature on biomechanical risk factors for knee OA. The Authors' are encouraged to emphasize the confirmatory and public health implications more explicitly in the discussion.

Authors' response
Thank you for your comment. We agree that our findings largely confirm previous evidence on biomechanical risk factors for knee OA. As suggested, we have now strengthened the discussion to more explicitly highlight the confirmatory nature of the results and their public health implications, particularly the relevance for prevention strategies in the construction industry.

Authors' action
The following sentences have been added to the Discussion section (page 20):
"*The results are confirmatory in nature and support existing evidence on biomechanical exposures and OA-related arthroplasty. These findings have important public health implications, particularly for developing targeted prevention strategies in the construction industry.*"

And the Conclusion (page 20) is now written as:

"In conclusion, our findings ~~provide further~~ *confirm and extend existing* evidence that occupational workload is associated with an increased risk of knee arthroplasty due to OA."

Reviewer 1 Comment:
2. The null findings for hip OA could benefit from clearer interpretation. It should be specified whether this is due to insufficient statistical power or reflects true etiological differences.

Authors' response
We agree that the interpretation of the null findings for hip OA needs clarification. Given the large number of hip arthroplasty cases, insufficient statistical power is unlikely to explain the absence of associations. Instead, the consistent null results across exposure measures suggest possible etiological differences, where occupational biomechanical load appears more relevant for knee OA than for hip OA.

Authors' action
The following sentences have now been added to the Discussion section:
"*Given the large number of cases with hip arthroplasty, insufficient statistical power is unlikely to explain the lack of associations with biomechanical exposures. Instead, this consistent absence across exposure measures suggests possible etiological differences, with occupational biomechanical load appearing more relevant for knee OA than for hip OA*."
In a previous study from the C*onstruction Workers Cohort*, with follow-up time until 1998 there were no statistically significant risks for hip arthroplasty in any of the occupational groups [15].

Reviewer 1 Comment:
3. Occupational titles, BMI, and smoking status were captured only at baseline. The implications of assuming stable exposure over decades should be addressed more thoroughly in the limitations.

Authors' response
Thank you for the opportunity to clarify. Occupational titles, BMI and smoking status were only available at baseline and could not be updated during follow-up due to data access constraints. We have now expanded the limitation section to further discuss the implications

of assuming stable exposures over time. Specifically, we acknowledge that BMI and smoking may change during adulthood; however, evidence shows that many adults' follows relatively stable BMI trajectories over decades, and persistent behaviour in smoking status.

Furthermore, in the original version of the manuscript already included a discussion of the lack of updated job titles information during follow-up, as well as our assumption – supported by previous research - that construction workers typically stays in the same occupational group. This discussion is retained in the current version. In addition, we have now further developed the limitation section regarding the variables BMI and smoking.

Authors' action

The follow changes have been made to the limitation section, and sentences has been added: "Regarding not updating information on occupational group, BMI and smoking status during follow-up, the following information has been added to the limitation section (page 19): "Furthermore, data on job title, BMI and smoking habits were collected during the health examinations and not updated during follow-up. This *may have resulted in exposure misclassification and required the* assumption that workers remained in the same occupational group *and that BMI and smoking habits stayed relatively stable over time. Although personal factors such as BMI and smoking may change, longitudinal studies show substantial tracking of BMI across adulthood and persistence in smoking behavior [33-34].* A previous Swedish study also indicates that construction workers generally remain within the same occupational group [19], *suggesting that changes in biomechanical exposure due to job mobility are limited. Any resulting misclassification is likely non-differential and would bias the results toward the null.*"

Reviewer 1 Comment:

4. The manuscript states that Poisson regression was used to estimate relative risks (RR). To avoid confusion with prevalence ratios, especially among general readers, the authors may consider clarifying that the model estimates incidence rate ratios (IRR) based on person-time data in a longitudinal cohort.

Authors' response and action

Thank you for your comment. We agree and have now revised the manuscript accordingly throughout. For example, see the Statistical Analysis section (page 8).

"Poisson regression models were utilized to calculate incidence rate *ratios* (IR*R*), wherein the counts of hip arthroplasty due to OA and person-years at risk were compared across levels of biomechanical exposures, ~~including the logarithm of person-years at risk as offset.~~ *These IRRs were interpreted as representing the relative differences in incidence rates of hip surgery across exposure categories rather than prevalence ratios.*

Reviewer 2, specific comment, (Dr. Ahmet OZTERMELI, Gebze Fatih State Hospital)

Comments to the Author:
Given the small magnitude of the observed risk increases, it is unclear whether these findings are clinically meaningful.
Occupational exposure, BMI, and smoking status were assessed only at the first health examination and were not updated during follow-up. This may have resulted in exposure misclassification and should be acknowledged more clearly as a limitation.
Arthroplasty reflects severe osteoarthritis but is also influenced by factors such as healthcare access, patient preferences, and surgical decision-making. These factors should be acknowledged as potential sources of bias.

Authors' response
Thank you for your comments and providing us with the opportunity to clarify. We agree that the observed risk increases are modest. Nonetheless, even small relative increases may have relevance at the population level in large occupational groups with long-term and repeated exposure to heavy biomechanical loads. We have added this perspective to the Discussion section to avoid overstating the clinical implications.

Regarding your second point, we acknowledge that occupational group, BMI and smoking status were assessed only during the health examinations and were not updated during follow-up. This may have resulted in exposure misclassification; however BMI and smoking tend to track across adulthood, and most construction workers remain in similar occupational roles. Any misclassification is therefore likely non-differential and would bias the results toward the null. We have now expanded the limitation accordingly.

Regarding factors influencing arthroplasty – this is a section that we previously have touched upon in the Discussion section: "It is also essential to recognize that the process leading to arthroplasty is multifactorial, incorporating individual willingness to undergo surgery and socioeconomic factors [28]. Additionally, surgeons may be less inclined to operate on patients with physically demanding occupations.", which we now have moved to limitations, expanded and acknowledged as a potential source of bias.

Authors' action
Regarding clinically meaningful – we have added the following information in the Discussion section (page 16): "Although, the incidence rate ratios and their upper confidence interval *limits* were modest, they consistently indicated an increased risk, *and even small relative increases may have an impact in large occupational groups with long-term and repeated exposures to heavy biomechanical loads.*"

Regarding not updating information on occupational group, BMI and smoking status during follow-up, the following information has been added to the limitation section (page 19): "Furthermore, data on job title, BMI and smoking habits were collected during the health examinations and not updated during follow-up. This *may have resulted in exposure misclassification and required the* assumption that workers remained in the same occupational group and that BMI *and smoking habits stayed relatively stable over time. Although personal factors such as BMI and smoking may change, however longitudinal studies show substantial tracking of BMI across adulthood and persistence in smoking behavior [33-34].* A previous Swedish study also indicates that construction workers

generally remain within the same occupational group [19], suggesting that changes in biomechanical exposure due to job mobility are limited. *Any resulting misclassification is likely non-differential and would bias the results toward the null.*"

Regarding how factors affecting the process of surgery decision; we now acknowledge this as a potential source of bias in the limitation section (page 19):

"It is also essential to recognize that the process leading to arthroplasty is multifactorial, incorporating individual willingness to undergo surgery, socioeconomic factors, [28] *access to healthcare and surgeons' decision-making. These factors may affect the likelihood of receiving arthroplasty and therefore represent a potential source of outcome misclassification and bias.*"

Reviewer 3, specific comment, (Prof. Zoe Kelson, University of Exeter)
Comments to the Author:
This study aims to evaluate the association between exposure to occupational biomechanical factors and the incidence of surgically osteoarthritis (OA) treated with arthroplasty in the hip and knee among male construction workers.

Reviewer 3 Comment:
"This longitudinal register-based cohort study followed male construction workers for 33 years to identify arthroplasties in the hip or knee due to OA and to explore the relationship between prior occupational biomechanical exposures, and the incidence of surgery"
The authors do well to utilise a large longitudinal dataset in this study.

"Male construction workers (n=291 062) who participated in a national Swedish occupational health examination program between 1971 and 1993 were followed from 1987 to 2019"
and
"Two outcome variables were utilized in this study: the first occurrence of surgically treated hip OA and knee OA, each assessed separately during the follow-up period from 1987 to 2019. "
Can the authors please consider whether follow-up data can be updated to more recent years?

Authors' response
While it is theoretically possible to extend the follow-up beyond 2019, doing so would require a new data extraction from the National Patient Register, including an amendment to the existing ethical approval and a renewed application to the data holder (the National Board of Health and Welfare). This process involves substantial administrative steps, additional costs, and processing times that can extend to over a year. Consequently, an update is not feasible within the timeline of the current manuscript, but we will consider it for future projects on the Construction Workers Cohort.

Authors' action
None

Reviewer 3 Comment:
"The study included 10 336 cases of hip arthroplasties and 8926 cases of knee arthroplasties" [Abstract]
and
"The study cohort was selected from a total of 389 132 Swedish construction workers who participated in health examinations as part of a national health surveillance program conducted from the late 1960s until 1993 (the Construction Workers Cohort). The construction workers were offered free, voluntary health checks through agreements established between employers and labor unions, with approximately 80% completing at least one health examination"
and
"In the study cohort, we included male construction workers examined between 1971 and 1993. We used data from each individual´s first health examination, including those aged 15 to 67."

Can the authors please further comment on whether the included cohort can be considered to be representative?

Authors' response
Thank you for this important comment. The study cohort derives from the nationwide "Construction Workers Cohort", in which approximately 80% of all Swedish construction workers underwent at least one health examination during the national health surveillance program. Given this high participation rate and the fact that the health examinations were offered free of charge as part of union-employer agreements, we consider the underlying cohort broadly representative of construction workers from this period.

For the current study, we restricted the cohort to male construction workers because women constituted a very small proportion of the original cohort and were predominantly employed in white-collar roles. Including them would have introduced heterogeneity not relevant to the occupational exposures assessed. Furthermore, we applied an upper cut-off of 40 kg/m^2 rather than 35 kg/m^2 as used in previous studies on this cohort, to better reflect contemporary surgical eligibility thresholds, as described in the Method section at page 6. Despite these restrictions, we considered the analytic sample representative of *Swedish male construction workers.*

Authors' action
In the Discussion section (page 20), the following sentences have been added: "To the best of our knowledge, this is the largest study conducted to date on biomechanical risk factors for hip and knee arthroplasty. *It is based on a large national cohort that is highly representative of Swedish male construction workers, and covers an extended follow-up period, which strengthens the validity of the findings.*"

Reviewer 3 Comment:

Can cohort demographics please be tabulated?

Authors' response
Thank you for comment. We have chosen to present the cohort demographics in running text in the first paragraph of the Result section (page x).

Authors' action
None.

Reviewer 3 Comment:
"Individuals with missing height or weight data, those who were unusually short (<150 cm) or tall (>200 cm), and those with a BMI outside the range of 17 to 40 kg/m 2 were excluded... Furthermore, individuals not able to classify in a specific occupational group were excluded, since they could not be mapped according to the JEM (Figure 1)."
Can the authors please assess whether missing data can be considered to be missing at random?

Authors' response
Thank you for the opportunity to clarify. Missing height and weight were rare and mainly due to incomplete examination records. A dropout analysis showed that excluded individuals were on average, 2.6 years younger and 3 cm shorter than those included, indicating that the missingness can be explained by observed characteristics and therefore consistent with Missing at Random (MAR).

Additionally, the systematic removal of workers performing ”Other work” who could not be mapped in the JEM, indicates that the missingness arises from observed, structural reason and is therefore compatible with Missing at Random (MAR).

Authors' action
None.

Reviewer 3 Comment:
"Data on age, smoking habits, body mass index (BMI), job title, and self-reported biomechanical exposures were collected during the health examinations. " [Abstract]
and
"Data on smoking habits (classified as never, ever, or unknown) and height and weight, from which BMI was calculated, were collected from each individual´s initial health examination. Age at the health examination was also recorded. "

Can the authors please confirm if there was any other missing data (e.g. age or BMI)?
If so, how was this missing data handled in the analysis?

Authors' response

Missing data of smoking habits was classified as unknown as described in the manuscript, there was no missing data regarding age and regarding BMI – those who had missing data on height or weight was excluded from the analyses at enrolment (please see Figure 1 – flowchart).

Authors' action
No further action.

Reviewer 3 Comment:
"Each individual's job title was collected from their last registered health examination." Can the authors please clarify how changes in job and occupation are handled in the analysis?

Authors' response
It is correct that we used each individual´s job title from their last registered health examination in the analyses. We did not have access to information on job or occupational changes during the follow-up period. Therefore, our analyses relies on the assumption that workers remained within the same occupational group during the observation period.

We have acknowledged this as a methodological limitation and have now further developed this section of the manuscript.

Authors' action
This section of the limitation now discuss this matter:

"Furthermore, data on job title, BMI and smoking habits were collected during the health examinations and not updated during follow-up. This *may have resulted in exposure misclassification and required* the assumption that workers remained in the same occupational group *and that BMI and smoking habits stayed relatively stable over time. Although personal factors such as BMI and smoking may change, however longitudinal studies show substantial tracking of BMI across adulthood and persistence in smoking behavior [33-34]*. A previous Swedish study also indicates that construction workers generally remain within the same occupational group [19], *suggesting that* changes in biomechanical exposure *due to job mobility are limited. Any resulting misclassification is likely non-differential and would bias the results toward the null."*

Reviewer 3 Comment:

"Between 1989 and 1993, an additional questionnaire was used during the health examinations to gather data on self-reported exposure to various biomechanical exposures."

Can the authors please acknowledge the potential for self reporting bias, for these additional questions as well as other variables in the analysis?

Authors' response
Thank you for this comment. We acknowledge the potential for self-reporting bias in the additional questionnaire with self-reported exposure. Self-reported biomechanical exposures

may be influenced of symptoms, prior knowledge of diseases, or expectations about risks, which could lead to over- or underestimation of exposures. We have now expanded the limitation section to explicitly address this issue. Importantly, in our study, results based on self-reported exposures were consistent with those obtained using the JEM-based exposure assessments, which reduces the concern that self-reporting bias substantially affected our conclusions.

Furthermore, smoking habits were also self-reported.

Authors' action
The following information has been added in the limitation section of the manuscript: "In a subgroup of the cohort, we also *analyzed* self-reported exposure data. *Self-reported information may introduce bias, for example if individuals over- or underestimate exposures in relation to symptoms or perceived risk [22]. However, the consistency between results obtained from the self-reported data and the JEM-based exposure assessments for both hip and knee arthroplasty–* strengthens the *validity of our findings and suggests that self-reporting bias is unlikely to have materially influenced* the overall conclusions."

Regarding self-reported smoking habits, the following information has now been included in the Method section: "Data on smoking habits (*self-reported* - classified as never, ever, or unknown).." (page 6)

Reviewer 3 Comment:
"Poisson regression was applied to estimate adjusted relative risks (RR) associated with each type of occupational biomechanical exposure" [Abstract]
and
"Poisson regression analyses were conducted, designating biomechanical exposure as the primary independent variable and hip arthroplasty due to OA as the outcome variable...
Poisson regression models were utilized to calculate incidence rates (IR), wherein the counts of hip arthroplasty due to OA and person-years at risk were compared across levels of biomechanical exposures, including the logarithm of person-years at risk as offset...
All regression analyses were adjusted for age at follow-up and BMI at health examination (both modelled using restricted cubic splines with four knots positioned at the 5th, 35th, 65th, and 95th percentiles), smoking status, and the calendar year as a linear effect. Poisson regressions were performed for each of the biomechanical exposures derived from the JEM and for the self-reported biomechanical exposures (among the subgroup that responded to the questionnaire). All analyses were repeated using knee arthroplasty as the outcome"
Appropriate modelling methods have been applied by the authors.

Can the authors please expand on the description of the model, including how time-varying confounders were included as covariates and clarifying how the longitudinal data was analysed?

Authors' response
A Lexis expansion was used to transform the cohort into a person-year dataset, in which each participant contributed one observation for each year of follow-up. This allowed us to

correctly account for covariates that change over time. Specifically, age and calendar year was updated for each one-year interval. Thus, both age and calendar year entered the regression models as time-varying covariates. In contrast, BMI and smoking status, which were measured at the baseline health examination, were included as fixed covariates. This structure ensured that the Poisson regression models appropriately reflected the longitudinal nature of the data.

Authors' action
We have now added this clarification to the Methods section (page 8).

"A Lexis expansion was applied to split each participant's follow-up into consecutive one-year intervals. Age and calendar year was recalculated and updated at the start of each interval. These variables were treated as time-varying covariates in the Poisson regression models, accounting for the time-varying effects of age and calendar year throughout the study period."

Reviewer 3 Comment:
"The follow-up period began in the year after the initial health examination, on January 1, 1987, or in the year the individuals reached 40, whichever occurred later.
Observations were censored at instances of death, emigration, reaching the age of 80 years, or at the conclusion of the study period at December 31, 2019"
Can the authors please clarify if and how differences in follow-up time have been adjusted for in the analyses?

Authors' response
Yes. Differences in follow-up time were accounted for by including the logarithm of person-years at risk as an offset term in the Poisson regression models. Adjustments were made for age and calendar year. The offset ensured that individuals contributed risk time according to their actual duration of follow-up.

Authors' action
None

Reviewer 3 Comment:
Did the authors please consider applying a Cox regression, given the time-to-event nature of the study?

Authors' response
In our cohort data, we need to model two different time scales, age and calendar time, due to variations in incidence across both. Therefore, we have divided the data into one-year intervals. This approach assumes that the incidence within each time interval is constant, but this assumption is relaxed by using relatively short intervals and applying smoothing curves from splines to adjust for the time scales. The main advantage of this method compared to traditional Cox regression is that we can account for both age and calendar time, rather than

modelling only one time scale, as in traditional Cox regression. Additionally, Cox regression can be considered a special case of Poisson regression[2]. Each individual's follow-up is still censored for death and emigration.

References:
1) Batyrbekova, Nurgul, et al. "Modelling multiple time-scales with flexible parametric survival models." BMC medical research methodology 22.1 (2022): 290.

2) Carstensen, Bendix, Who needs the Cox model anyway? Version 4 (2023). Retrieved from https://bendixcarstensen.com/WntCma.pdf [2025-01-13]

Authors' action
None.

Reviewer 3 Comment:
"By including only male construction workers, the study´s findings may not be generalizable to women." [Strengths and Limitations]
and
"Some methodological considerations should be addressed..."
The authors provide a Discussion on the study limitations and assumptions.
Thanks for providing a copy of the STROBE checklist.

Authors' response
Thank you for your careful review of our manuscript, providing us with the opportunity to clarify and motivate our choices and further strengthen the manuscript.

VERSION 2 - REVIEW

**Reviewer**	**1**
**Name**	**Kang, Mo-Yeol**
**Affiliation**	**College of Medicine, The Catholic University of Korea, Department of Occupational and Environmental Medicine**
**Date**	**13-Mar-2026**
**COI**	

The authors have addressed all comments and made appropriate revisions and corrections to the revised manuscript. The revised one is much improved so that I have no more comments to be revised for the article.

**Reviewer**	**2**
**Name**	**OZTERMELI, Ahmet**
**Affiliation**	**Gebze Fatih State Hospital, Ortopedics and Traumatology**

Date **18-Mar-2026**

COI

The authors have adequately addressed the comments raised in the previous review and revised the manuscript accordingly. The revisions are reasonable and improve the clarity of the study. I recommend acceptance of the manuscript in its current form.
